# Hybrid genome assembly of colistin-resistant *mcr-1.5*-producing *Escherichia coli* ST354 reveals phylogenomic pattern associated with urinary tract infections in Brazil

**DOI:** 10.1016/j.jgar.2024.02.017

**Published:** 2024-06

**Authors:** Bruna Fuga, Fábio P. Sellera, Fernanda Esposito, Quézia Moura, Marcelo Pillonetto, Nilton Lincopan

**Affiliations:** a Department of Microbiology, Instituto de Ciências Biomédicas, Universidade de São Paulo, São Paulo, Brazil; b Department of Clinical Analysis, Faculdade de Ciências Farmacêuticas, Universidade de São Paulo, São Paulo, Brazil; c One Health Brazilian Resistance Project (OneBR), Brazil; d Department of Cell Biology, Institute of Biological Sciences, University of Brasília, Brasília, Brazil; e Department of Internal Medicine, School of Veterinary Medicine and Animal Science, University of São Paulo, São Paulo, Brazil; f School of Veterinary Medicine, Metropolitan University of Santos, Santos, Brazil; g Federal Institute of Espírito Santo, Vila Velha, Brazil; h Postgraduate Program in Infectious Diseases, Federal University of Espírito Santo, Vitória, Brazil; i State Public Health Laboratory of Paraná, São José dos Pinhais, Brazil; j Pontifical Catholic University of Paraná, Curitiba, Brazil

**Keywords:** *Enterobacterales*, Plasmid-mediated colistin resistance, Plasmidome, Resistome, Phylogenomics

## Abstract

**Background:**

The rapid and global spread of *Escherichia coli* carrying *mcr*-type genes at the human-animal-environmental interface has become a serious global public health problem.

**Objective:**

To perform a genomic investigation of a colistin-resistant *E. coli* strain (14005RM) causing urinary tract infection, using a hybrid de novo assembly of Illumina/Nanopore sequence data, presenting phylogenomic insights into the relationship with *mcr-1*-positive strains circulating at the human-animal-environmental interface, in Brazil.

**Methods:**

Genomic DNA was sequenced using both the Illumina NexSeq and Nanopore MinION platforms. De novo hybrid assembly was performed by Unicycler. Genomic data were assessed by in silico prediction and bioinformatic tools.

**Results:**

The genome assembly size was 5 333 039 bp. The *mcr-1.5*-positive *E. coli* strain 14005RM belongs to the sequence type ST354 and presented a broad resistome (antibiotics, heavy metals, disinfectants, and glyphosate) and virulome. The *mcr-1.5* gene was carried by an IncI2 plasmid (p14005RM, sizing 65,458 kb). Full genome SNP-based phylogenetic analysis reveals that *mcr*-1.5-producing *E. coli* strain 14005RM is highly related (> 98% identity) to colistin-resistant *mcr*-1.1-positive ST354 lineages associated with urinary tract infections in Brazil since 2015.

**Conclusion:**

Mobile colistin resistance within the Brazilian One Health microbiosphere is mediated by *mcr* gene variants propagated by IncX4, IncHI2, and IncI2 plasmids, circulating among global clones of *E. coli*.

## Highlights

• Colistin-resistant *E. coli* carrying *mcr*-type genes have spread rapidly worldwide.
• *E. coli* ST354 carrying *mcr-1.5*/IncI2 from urinary tract infection is reported in Brazil.
• Phylogenomic cluster of ST354 associated with urinary tract infections is highlighted.
• The rapid adaptation of *mcr*-positive *E. coli* within a One Health context is discussed.

## 1 Introduction

Polymyxins are last-resort antimicrobial agents usually reserved to treat infections caused by multidrug-resistant (MDR) Gram-negative pathogens resistant to all the other currently available antibiotics [1,2]. Critically, the mobile phosphoethanolamine transferase *mcr-1* gene, responsible for transferable colistin resistance, was first reported in 2016, in China [3], and then other *mcr* alleles, including *mcr-2, mcr-3, mcr-4, mcr-5, mcr-6, mcr-7, mcr-8, mcr-9*, and *mcr-10* have been described in *Enterobacterales* worldwide [4,5].

Specifically, in Brazil, the occurrence of *Escherichia coli* carrying *mcr-*type genes has been reported in humans [6], [7], [8], [9], [10], food (chicken meat) [10], [11], [12], aquatic environments [10,^13^,14], farm and food-producing animals [10,^15^,16], and wildlife [10,17]. Herein, we present genomic insights into an *E. coli* ST354 carrying an IncI2 plasmid-mediated *mcr-1.5* gene isolated from a human patient. We also investigated its phylogenomic relatedness with other Brazilian *mcr-1*-positive strains circulating at the human-animal-environment interface.

## 2 Materials and methods

In January 2017, a patient was admitted to a hospital in South Brazil with a urinary tract infection. A urine sample was collected and then submitted to urine culture. One *E. coli* isolate was recovered from the urine sample, identified by matrix-assisted laser desorption ionisation–time of flight mass spectrometry (MALDI-TOF MS) analysis, and further confirmed by whole genome sequencing (WGS) analysis. *E coli* strain 14005RM was subjected to an antimicrobial susceptibility test by the disk-diffusion method following the recommendations of the Clinical and Laboratory Standards Institute - CLSI Supplement M100, 30th ed (https://clsi.org). Specifically, breakpoints used for enrofloxacin and ceftiofur were obtained from supplement VET08 (CLSI supplement VET08, fourth ed). Moreover, colistin susceptibility was determined by the broth microdilution method according to European Committee on Antimicrobial Susceptibility Testing (EUCAST) guidelines (http://www.eucast.org/ast_of_bacteria/warnings/#c13111).

Genome sequencing was carried out on the Illumina PE NextSeq platform (San Diego, USA), and MinION sequencer (Oxford Nanopore Technologies, Oxford, UK). For Illumina, total genomic DNA was extracted using a PureLink quick gel extraction kit (Life Technologies, CA). Subsequently, genomic DNA was used to library construction with a Nextera DNA Flex Kit (Illumina, San Diego, CA). For Nanopore sequencing, genomic DNA was extracted using the MasterPure Complete DNA and RNA Purification Kit (Lucigen) and the Nanopore Rapid Barcoding Sequencing Kit (SQK-RBK004; Oxford Nanopore, Oxford, UK) was used for library construction. Total DNA was sequenced with an R9.4.1 MinION flow cell (FlO-MIN106) for a 48h run using MinKNOW v.19.10.1 software.

The short reads were initially subjected to a quality check using FastQC software (http://www.bioinformatics.babraham.ac.uk/projects/fastqc), and the paired reads were trimmed to remove adapters and low-quality regions (with a PHRED quality score below 20) using TrimGalore v0.6.5 (https://github.com/FelixKrueger/TrimGalore). For long-read basecalling and to trim barcode and adapter sequences, Guppy v3.3.3 software was used. Additionally, Filtlong v0.2.0 (https://github.com/rrwick/Filtlong) was used to filter long reads based on quality, employing the default parameters of –min_length 1000 (discarding reads shorter than 1 kbp); –keep_percent 90 (eliminating the worst 10% of read bases); –trim (trimming bases from the start and end of reads that do not match a k-mer in the reference); and –split 500 (splitting reads when 500 consecutive bases fail to match a k-mer in the reference).

De novo hybrid assembly was performed using Unicycler v0.4.8 (https://github.com/rrwick/Unicycler), and visualised using the Bandage assembly graph viewer v0.8.1 (https://github.com/rrwick/Bandage). Annotation was performed with NCBI PGAP v.3.2 (http://www.ncbi.nlm.nih.gov/genome/annotation_prok/).

The genome was analysed to identify antimicrobial resistance genes (ARGs), chromosomal point mutation, multilocus sequence typing (MLST), plasmid replicons, virulence genes, species confirmation, serotype, and *fim* type using multiple databases available from the Center for Genomic Epidemiology (http://genomicepidemiology.org/). The phylogroup of the *E. coli* 14005RM strain was determined by ClermonTyping v1.4.0 (https://github.com/A-BN/ClermonTyping). The VFDB (https://github.com/haruosuz/vfdb) database was also used for virulome prediction. Additionally, heavy metal- (HM), pesticide- (glyphosate), and disinfectant- (QACs) resistant genes were analysed by ABRicate v0.9.8 (https://github.com/tseemann/abricate) using a database constructed from NCBI and BacMet2 (http://bacmet.biomedicine.gu.se/) genes. For all predicted genes, a ≥90% identity and ≥80% coverage threshold were used.

For investigations of the phylogenetic relationship between *E. coli* 14005RM and other *mcr*-1 variants of *E. coli* strains from Brazil, we used the available genomes deposited in the NCBI GenBank database (https://www.ncbi.nlm.nih.gov/genbank/). Genomes were annotated by Prokka (https://github.com/tseemann/prokka). We used Roary v3.13.0. (https://github.com/sanger-pathogens/Roary) to deduce the group of genes (core genome) shared by the colistin-resistant *mcr-1.5-*producing *E. coli* ST354 and the 28 related *E. coli* strains of interest. A multi-FASTA alignment of all of the core genes was created, and SNPs found in the genes in the core genome (core genome SNPs) were used to infer relationships between the strains. In this regard, SNP sites was used for assessing polymorphic sites (https://github.com/sanger-pathogens/snp-sites), and SNP-Dists was used to construct an SNP distance matrix (https://github.com/tseemann/snp-dists) providing the number of single nucleotide polymorphisms between each pair of isolates in the alignment. In brief, our analysis focused on SNP variations of a core genome shared among isolates. A limitation of the methodology was it not being possible to verify deletions.

Subsequently, a maximum likelihood tree, tested against 100 bootstrap replications, was constructed using RAxML-NG (https://github.com/amkozlov/raxml-ng) v0.9.0 (model GTR+G). The tree was visualised with iTOL (https://itol.embl.de/) v5.6.1).

The Mlplasmids tool (https://sarredondo.shinyapps.io/mlplasmids/) was used to predict plasmid and chromosome-derived sequences, respectively. The *mcr-1.5*-containing plasmid was compared with other sequences using the NCBI BLASTn database and Geneious software. Additionally, the plasmid image was generated using EasyFig (default parameters) (https://mjsull.github.io/Easyfig/).

## 3 Results and discussion

*E. coli* strain 14005RM displayed a resistance profile to colistin (4 mg/L), nalidixic acid, ciprofloxacin, enrofloxacin, gentamicin, sulfamethoxazole/trimethoprim, chloramphenicol, and tetracycline (Supplementary Table S1), remaining susceptible to meropenem, ertapenem, imipenem, ceftriaxone, ceftazidime, cefoxitin, cefepime, cefotaxime, amoxicillin/clavulanic acid, aztreonam, amikacin, fosfomycin, cephalothin, ceftiofur, and ampicillin.

From Illumina sequencing, a total of 2,901,194 reads were generated, with an average read length of 75 bases, resulting in a cumulative sequence length of 291,150,004 nucleotides. In contrast, Nanopore sequencing yielded 119,978 reads, with a total of 854,994,143 nucleotides and a read length ranging from 110 to 102,758 bases (median read length of 3,984). After filtering, a total of 34,007 reads and 500,000,984 nucleotides, along with a 11,699 median read length, were obtained.

The genome size of *E. coli* 14005RM after hybrid assembly was 5,333,039 bp, comprising 23 contigs with a GC content of 50.4%. The NCBI PGAP annotation identified 4832 protein-coding genes.

In silico analysis based on MLST, Clermont typing, serotype, and *fimH* subtyping revealed that *E. coli* strain 14005RM belonged to ST354, phylogroup F, O153:H34, and *fimH38*, respectively. The ST354 has been globally recovered from human, animal, soil, and raw vegetable samples [8,^9^,[18], [19], [20], [21], [22], [23], [24], [25]], supporting its potential for dissemination and adaptation to different settings and representing a One Health concern. Noteably, previous studies also reported phylogroup F *E. coli* ST354 causing human bloodstream and urinary tract infections (UTIs) in China [21] and Brazil [22], as well as UTIs in a dog in Thailand [23]. In this regard, the high ability to infect both the bladder and the kidney of phylogroup F isolates has also been reported [20].

Virulome investigation revealed the presence of genes related to autotransporter (*air*), adherence factors (*fimB, fimE, fimI, fimC, fimD, fimF, yfcV, lpfA, hra, ecpR, ecpA, ecpB, ecpC,* and *ecpD*), invasion (*ibeA*), specific uropathogenic protein (*usp*), bacteriocins (*cib*), iron acquisition systems (*shuA, chuA, chuT, chuW, chuU, chuV, fepA, fepB, fes, entB,* and *entE*), proctin/serum resistance (*kpsE, kpsM_K15*, and *kpsD*), and others (*gad, eilA*).

ST354 has also being associated with *mcr*-type and/or ESBL production [8,^9^,^18^,19]. In fact, through resistome analysis of 14005RM isolate, several genes encoding resistance have been found, including to colistin (*mcr-1.5*), aminoglycosides (*ant(2”)-Ia, aadA1, aadA2*), macrolides (*mdf(A)*), trimethoprim (*dfrA12*), phenicol (*cmlA1*), and sulphonamide (*sul1, sul3*). Nucleotide substitutions in the *gyrA* (S83L and D87N), *parC* (S80I and E84G), and *parE* (I355T) genes were identified through genome sequencing, resulting in changes in the predicted amino acid sequence. Additionally, genes conferring resistance to heavy metals (*merR*), disinfectants (*qacE, qacF, tehA, tehB, emrD, acrE, tolC,* and *mdtE*), and glyphosate (*phnL, phnE,* and *phnC*) were identified.

**Fig. 1:**
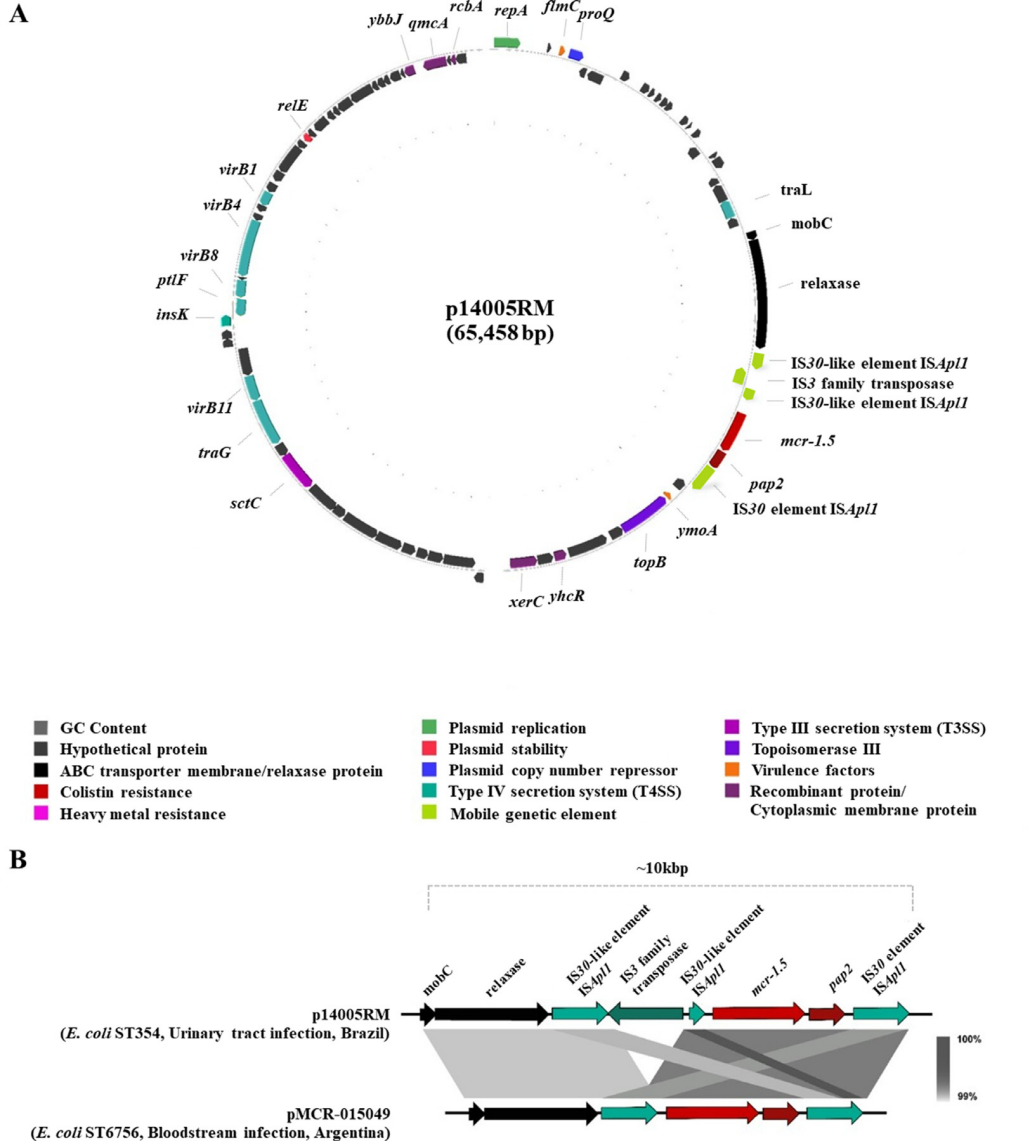
Circular representation of plasmid harbouring *mcr-1.5* in *Escherichia coli* strain 14005RM. (A) A schematic representation of genes encoded by p14005RM plasmid showing colistin resistance in red, ABC transporter membrane/periplasmic binding protein in black, heavy-metal resistance in pink, plasmid replication in lime green, mobile-genetic elements in jade green, type IV secretion system in French lime green, type III secretion system in magenta, type IV conjugation system in jungle green, topoisomerase III in purple, virulence factors in orange, recombinant protein/cytoplasmic membrane protein in mulberry, and hypothetical proteins in dark grey. (B) The genetic context of *mcr-1.5* is highlighted in a linear view.

Hybrid genome assembly revealed that colistin-resistant *E. coli* strain 14005RM harboured the *mcr-1.5* gene on a circular p14005RM plasmid 65 kb in size (GenBank accession no. JAAWUF020000023.1) belonging to the IncI2 replicon type (Figure 1A). The p14005RM plasmid shared a very similar genetic environmental (identity: >99% and coverage: >94%) with the IncI2/*mcr-1.5* plasmid (pMCR-015049) from ST6756 *E. coli* previously identified in a human from Argentina (GenBank accession no. KY471308) [26] (Figure 1B). The *mcr-1.5* gene of the p14005RM plasmid was flanked upstream by *mobC* and *relaxase* genes, IS*30*-like element IS*Apl1*, IS*3* family transposase, and IS*30*-like element IS*Apl1*, while downstream, a *pap2* gene and IS*30* element IS*Apl1* were located (Figure 1B). Additionally, the *mcr-1.5* gene shared a very similar genetic environment to previously published genomes in the GenBank database (China: CP053720.1, CP053736.1, CP095857.1, CP041628.1, CP032892.1, and CP047002.1; Belgium: CP061906.1; and the Netherlands: LR882943.1). The IncFIA replicon type was also detected in the genome of *E. coli* strain 14005RM.

**Fig. 2:**
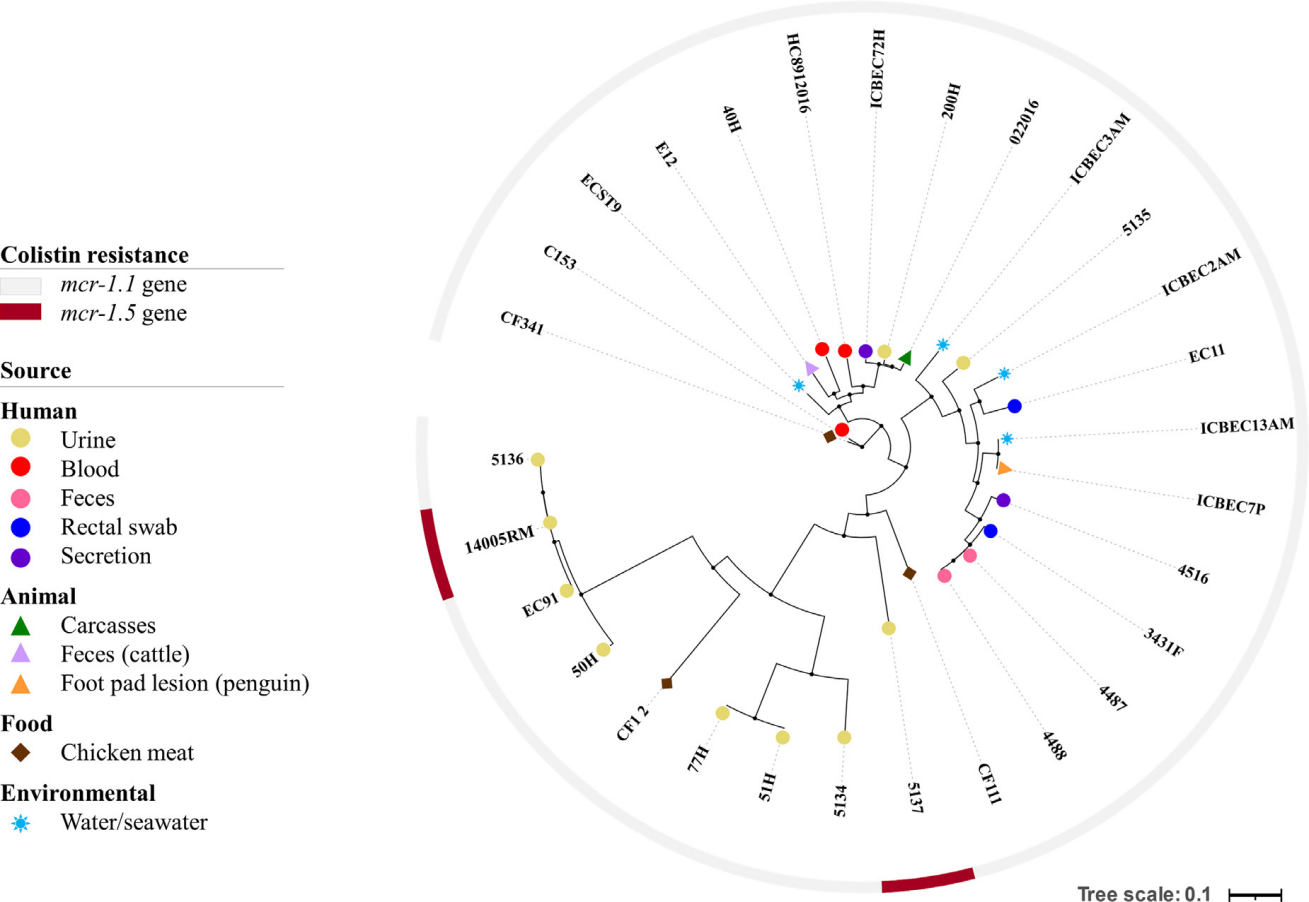
Phylogenetic relationship between *mcr*-1.5-producing *Escherichia coli* strain 14005RM (this study) belonging to the international clone ST354 and other 28 *E. coli* genomes that presented the *mcr*-1 gene from Brazil. The iTOL version 5.6.1 (https://itol.embl.de) was used to view the image.

For phylogenomic analysis, we selected 28 *E. coli* genomes from the NCBI database that harboured *mcr-1* variants, isolated from humans, animals, food, and natural environments in Brazil (Supplementary Table S2). A total of 2714 core genes were shared by all *E. coli* strains. The *E. coli* strains from distinct hosts and sources (i.e. humans, animals, food, and the environment) were closely grouped on the tree (Figure 2). Interestingly, this study reports the presence of the *mcr-1.5* gene in only two *E. coli* strains (i.e. 14005RM/ST354 and 5137/ST57; GenBank accession no. JAATKR000000000.1).

Phylogenomic analysis clustered (487, 818, and 2575 SNP differences) *E. coli* 14005RM with three *mcr-1.1*-positive *E. coli* strains isolated in 2015 (the EC91 strain) and 2017 (the 5136 and 50H strains), from patients with urinary tract infection, in South (Florianópolis city) and Southeast Brazil (São Paulo and Campinas cities), respectively (Supplementary Table S3).

In summary, we report the first draft genome sequence of an *E. coli* ST354 carrying an IncI2 plasmid-mediated *mcr-1.5* gene isolated from a human patient in Brazil. We also demonstrated that, although there is a certain degree of relatedness among the *mcr-1-*producing *E. coli* clones in Brazil, the diversity within the selected strains may be indicative of various factors influencing the genetic makeup, such as different hosts and sources, which may reflect its adaptability and versatility. Therefore, the identification of MDR *E. coli* carrying an IncI2 plasmid-mediated *mcr-1.5* gene among human clinical isolates represents a clinical challenge and an epidemiological alert that deserves continuous and effective surveillance. Considering the increasing rates of such pathogens globally, not only in human nosocomial settings but also outside hospitals, epidemiological genomic studies are in urgent demand. Finally, our data might provide additional information for comparative genomic analyses of molecular mechanisms, genetic structure, and epidemiological links of *mcr-1*-positive *E. coli* strains under the One Health umbrella.

## Appendix Supplementary materials

Image, application 1

Image, application 2

Image, application 3

Image, application 4
