# Supplementary material for: Hybrid genome assembly of colistin-resistant mcr-1.5-producing Escherichia coli ST354 reveals phylogenomic pattern associated with urinary tract infections in Brazil
Source: J Glob Antimicrob Resist. 2024 Jun;37:37–41. doi: 10.1016/j.jgar.2024.02.017 (PMC11183298; doi:10.1016/j.jgar.2024.02.017)
Supplement: Supplementary file 1 [file mmc1.docx]

**Supplementary Information**

**Table legends**

**Table S1**. Genetic and epidemiological characteristics of colistin-resistant *mcr-1.5*-positive *Escherichia coli* strain 14005RM belonging to the ST354.

**Table S2.** Genomic data of *mcr*-1-producing *Escherichia coli* strains from Brazil included in phylogenetic analyses.

**Table S3.** Matrix containing the pairwise number of SNP differences between all pairs of samples included in the analysis.
